# Kenny is the adaptor protein for ubiquitin-dependent mitophagy in *Drosophila melanogaster*

**DOI:** 10.1080/27694127.2026.2638025

**Published:** 2026-03-04

**Authors:** Hubert Osei Acheampong, Ryan Insolera

**Affiliations:** a Department of Ophthalmology, Visual and Anatomical Sciences, School of Medicine, Wayne State University, Detroit, MI, USA; b Graduate Program in Anatomy and Cell Biology, School of Medicine, Wayne State University, Detroit, MI, USA

**Keywords:** Kenny, mitophagy adaptor, optineurin, autophagy, phagophore, ALS, ubiquitin, autophagosome, VPS13D, mitochondria

## Abstract

Mitophagy is the selective degradation program for damaged and unnecessary mitochondria to maintain cellular mitostasis and survival. Specific mutations in the mediators for the canonical ubiquitin (ub)-dependent mitophagy pathway have been identified with unique neurological diseases like Parkinson disease and ALS (amyotrophic lateral sclerosis), metabolic diseases, and cancer. Mammalian OPTN (optineurin) has been shown as a SAR (selective autophagy receptor) for ub-dependent mitophagy in vitro with direct connections of its mutations with glaucoma and ALS. Despite the in vitro demonstration of OPTN’s role in mitophagy, the in vivo physiological characterization of OPTN’s mitophagy function is largely unexplored. In our recent study, we provide in vivo evidence that the *Drosophila melanogaster* (Dm) protein, Kenny, directly mediates the sequestration of target mitochondria for the progression and completion of ub-dependent mitophagy. This result establishes Kenny as the Dm homolog of OPTN. Previously, Kenny had only been characterized for its role in innate immune activation and modulation. The conclusion from this study provides avenues for further understanding the in vivo signaling regulating Kenny’s role in mitophagy and investigating homologous disease-relevant mutations of OPTN in Dm.

Ub-dependent mitophagy is a well-known process that is thought to proceed via the conventional PINK1 (PTEN-induced putative kinase 1)/PRKN/PARKIN (parkin RBR E3 ubiquitin protein ligase) pathway. In response to damaged mitochondria, PINK1 is stabilized on the OMM (outer mitochondrial membrane) and phosphorylates OMM proteins which lead to the recruitment and activation of the E3 ub ligase PRKN. PRKN promotes polyubiquitination of OMM proteins resulting in the procession of downstream mitophagy signals for the terminal clearance of the toxic mitochondria at the lysosome. Defective degradation of damaged mitochondria is implicated in neurodegenerative and cardiovascular diseases, partially because they are sources of toxic molecules like ROS. It took just over a decade to discover the intervening SARs that mediate the encapsulation of mitochondria by the phagophore for the progression of ub-dependent mitophagy in cell culture. The most characterized of these proteins are OPTN (optineurin) and NDP52/CALCOCO2 (Nuclear Dot Protein 52 kDa/Calcium binding and coiled-coil domain 2). OPTN is of special interest because of the critical association of its unique mutations with glaucoma and ALS. Despite the in vitro determination of OPTN as a SAR mediating mitophagy, it’s in vivo mitophagy function and physiological relevance is lacking. In our work, we showed that the Dm homolog of OPTN, Kenny, functions as ub-dependent SAR involved in mitophagy and is crucial for organismal survival under stressful mitochondria-damaging conditions [1].

Unlike the multiple SARs in mammals, the primary SAR recognized in Dm is Ref(2)p (refractory to sigma P), the homologue of SQSMT1/p62 (sequestosome 1). However, the Dm protein Kenny, previously determined to be the homolog of mammalian IKKγ (inhibitor of kappa B kinase gamma) with 23% identity and 35% similarity, is also homologous to mammalian OPTN with 19% identity and 36% similarity. We therefore sought to determine the role of these two candidate SARs in ub-mediated mitophagy in Dm. We first showed that ub-positive mitophagy intermediates accrued when we blocked basal mitophagy by removing the core autophagy gene *Atg5* (*Autophagy-related 5*) in larval motoneurons. This result was also observed when we knocked down (KD) the lipid transport gene, *Vps13D* (*vacuolar protein sorting 13D*), which we previously demonstrated led to stalled mitophagy intermediates containing enlarged ub-positive mitochondria, and incomplete phagophore formation around the mitochondria. Due to the prominent and better visualization of the mitochondria and the phagophore in the *Vps13D* RNAi model, we mainly utilized it for majority of our additional experiments. We next determined the localization of Ref(2)p and Kenny on the mitochondria in *Vps13D* KD to confirm their candidacy for a role as a ub-dependent SAR in mitophagy. We observed that both proteins accumulated on ub-positive mitochondria and thus may be involved in ub-dependent mitophagy. Since these intermediates were usually associated with phagophores, visualized via Atg8A (Autophagy-related 8A) staining, we probed the role of the potential SARs in inducing phagophore formation at ub-positive mitochondria. To answer this, we genetically removed Ref(2)p and Kenny separately in conditions of *Vps13D* KD and assessed whether the phagophores were still associated with the ub-positive mitochondria. Unlike in the Ref(2)p null mutants, recruitment of the phagophore to ub-positive mitochondria was disrupted in the Kenny null mutants. Knowing that the Kenny LIR (LC3-interacting region) is required to induce the local formation of the phagophore, we ablated this domain via the genomic engineering CRISPR/Cas9 technology. Similar to the Kenny null mutants, the phagophores associated with ub-positive mitochondria were largely reduced in the Kenny LIR mutant. These results established Kenny as essential to the degradation of ub-positive mitochondria by mitophagy.

We tested the role of Kenny for mitophagy in larval motoneurons using the end-stage mitophagy reporter, matrix-QC. There were no changes in basal mitophagy flux in both control and Kenny mutant conditions plausibly because of other active mitophagy pathways. We uncovered significant reduction in mitophagy in Kenny LIR and null mutants compared to WT control when we specifically induced ub-dependent mitophagy via *Drp1* (*dynamin-related protein 1*) KD in larval motoneurons. Similar reduction in mitophagy flux was observed when we induced ub-dependent mitophagy with FCCP (carbonyl cyanide *p*-(trifluoromethoxy) phenylhydrazone) in larval fat body in the Kenny null condition. We ascertained the role of Kenny for the survival of adult Dm under conditions of mitochondrial stress via paraquat feeding, of which Kenny null mutants had a significantly lower survival compared to WT control portraying Kenny’s importance in these conditions. A cross-species experiment was performed to determine if Kenny behaved comparable to OPTN in mammalian HeLa cells treated with CCCP (carbonyl cyanide m-chlorophenyl hydrazone) to induce mitophagy. Like with OPTN, Dm Kenny expressed in these mammalian cells was recruited together with PRKN to damaged mitochondria.

In conclusion, our results, summarized in Figure 1, demonstrate that Kenny is a ub-dependent SAR mediating mitophagy in Dm, akin to its mammalian homologue OPTN. Our discovery provides a platform to pursue the in vivo signaling cues needed for OPTN to draw the phagophore to mitochondria fated for ub-dependent mitophagy and examine what specific role Ref(2)p plays in executing this mitophagy. With the OPTN^E478G^ mutation associated with familial ALS, insight into its cell biology in Dm via its homologous mutation in Kenny will provide valuable steps toward understanding the pathogenesis of ALS. Overall, our study unravels a new pathway to uncover the specific signaling for Kenny-mediated mitophagy and its disease-relevant therapeutic applications.

**Figure 1.**
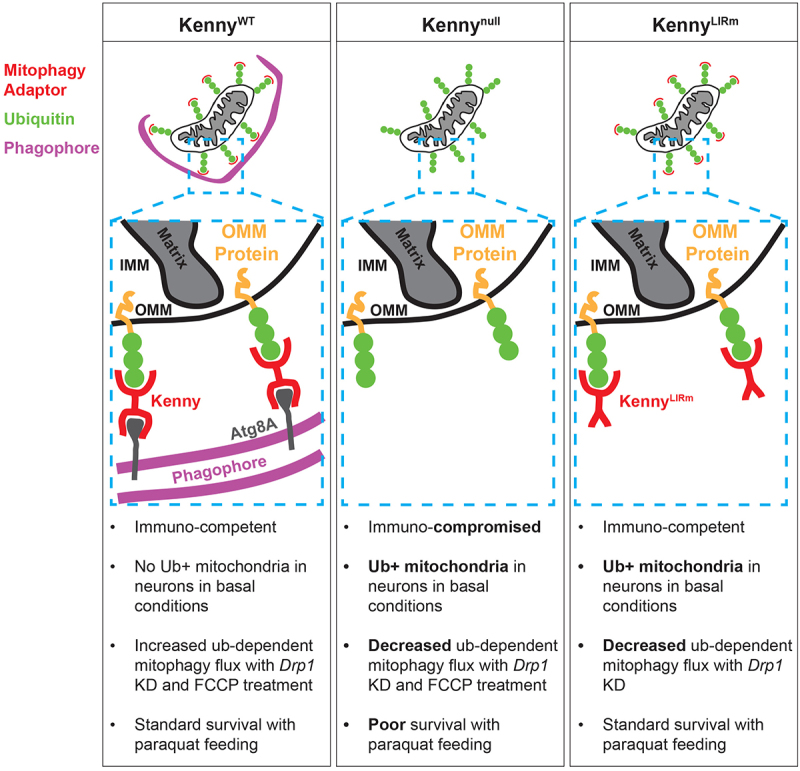
Schematic illustrations of the different Kenny genotypes generated to test the capability to induce phagophore formation at ub-positive mitochondria. The additional bulletins are representative summaries of the molecular and physiological consequences of the various Kenny modifications on ub-dependent mitophagy.

## Data Availability

Data sharing is not applicable to this article as no data were created or analyzed in this study.

## Abbreviations

ALS: Amyotrophic Lateral Sclerosis
Atg: Autophagy-related
CCCP: Carbonyl cyanide m-chlorophenyl hydrazone
CRISPR/Cas9: Clustered Regularly Interspaced Short Palindromic Repeats/CRISPR-associated protein 9
Dm: *Drosophila melanogaster*
Drp1: Dynamin-related protein 1
FCCP: Carbonyl cyanide *p*-(trifluoromethoxy) phenylhydrazone
IKK γ: inhibitor of kappa B kinase gamma
IMM: inner mitochondrial membrane
KD: knock down
LC3: Microtubule-associated protein 1A/1B-light chain 3
LIR: LC3-Interacting Region
matrix-QC: matrix quality control
NDP52/CALCOCO2: Nuclear Dot Protein 52 kDa/Calcium binding and coiled-coil domain 2
OMM: Outer Mitochondrial Membrane
PRKN: parkin RBR E3 ubiquitin protein ligase
PINK1: PTEN-induced putative kinase 1
Ref(2)p: Refractory to sigma P
ROS: Reactive Oxygen Species
SAR: selective autophagy receptor
SQSTM1/p62: Sequestosome 1
ub: ubiquitin
VPS13D: Vacuolar Protein Sorting 13D
WT: Wild type
